# CT-Based Radiomics Analysis to Predict Malignancy in Patients with Intraductal Papillary Mucinous Neoplasm (IPMN) of the Pancreas

**DOI:** 10.3390/cancers12113089

**Published:** 2020-10-23

**Authors:** David Tobaly, Joao Santinha, Riccardo Sartoris, Marco Dioguardi Burgio, Celso Matos, Jérôme Cros, Anne Couvelard, Vinciane Rebours, Alain Sauvanet, Maxime Ronot, Nikolaos Papanikolaou, Valérie Vilgrain

**Affiliations:** 1 Service De Radiologie, Assistance Publique-Hôpitaux De Paris, APHP. Nord, Hôpital Beaujon, 92110 Clichy, France; riccardo.sartoris@aphp.fr (R.S.); marco.dioguardiburgio@aphp.fr (M.D.B.); maxime.ronot@aphp.fr (M.R.); 2 Computational Clinical Imaging Group, Champalimaud Research, Champalimaud Foundation, Avenida Brasília, 1400-038 Lisbon, Portugal; joao.santinha@research.fchampalimaud.org; 3 Instituto de Telecomunicações, Instituto Superior Técnico, University of Lisbon, 1049-001 Lisbon, Portugal; 4 Centre De Recherche De L’inflammation (Cri), Inserm U1149, Université De Paris, 75018 Paris, France; 5 Radiology Department, Champalimaud Foundation, Avenida Brasília, 1400-038 Lisbon, Portugal; celso.matos@fundacaochampalimaud.pt; 6 Champalimaud Research, Champalimaud Foundation, Avenida Brasília, 1400-038 Lisbon, Portugal; 7 Service D’Anatomopathologie, Assistance Publique-Hôpitaux De Paris, APHP.Nord, Hôpital Beaujon, 92110 Clichy, France; jerome.cros@aphp.fr; 8 Service D’Anatomopathologie, Assistance Publique-Hôpitaux De Paris, APHP.Nord, Hôpital Bichat, 75018 Paris, France; anne.couvelard@aphp.fr; 9 Service De Pancréatologie, Assistance Publique-Hôpitaux De Paris, APHP.Nord, Hôpital Beaujon, 92110 Clichy, France; vinciane.rebours@aphp.fr; 10 Service De Chirurgie HPB, Assistance Publique-Hôpitaux De Paris, APHP.Nord, Hôpital Beaujon, 92110 Clichy, France; alain.sauvanet@aphp.fr; 11 Computational Clinical Imaging Group, Champalimaud Research, Champalimaud Foundation, 1400-038 Lisbon, Portugal; nickolas.papanikolaou@research.fchampalimaud.org

**Keywords:** risk stratification, quantitative imaging, texture, malignant transformation, radiomics, pancreatic cyst

## Abstract

**Simple Summary:**

The management of intraductal papillary mucinous neoplasms of the pancreas (IPMN) remains controversial due to the relatively high rate of unnecessary surgery for low grade dysplasia (LGD) despite the last international recommendations. The aim of our retrospective study was to assess the performance of radiomic analysis on CT in differentiating benign from malignant IPMN. We confirmed in a training cohort (296 patients) and a validation cohort (112 patients) that a total of 85 radiomics features provided valuable additional and independent information for discriminating benign from malignant tumors in the training cohort with an area under the ROC curve (AUC) of 0.84 and an external validation with an AUC of 0.71 with higher performance when implementing clinical variables leading to the indication to surgery. We have demonstrated the capabilities of radiomics models comprising LGD versus high-grade dysplasia (HGD) versus invasive, LGD and HGD, HGD and invasive.

**Abstract:**

To assess the performance of CT-based radiomics analysis in differentiating benign from malignant intraductal papillary mucinous neoplasms of the pancreas (IPMN), preoperative scans of 408 resected patients with IPMN were retrospectively analyzed. IPMNs were classified as benign (low-grade dysplasia, *n* = 181), or malignant (high grade, *n* = 128, and invasive, *n* = 99). Clinicobiological data were reported. Patients were divided into a training cohort (TC) of 296 patients and an external validation cohort (EVC) of 112 patients. After semi-automatic tumor segmentation, PyRadiomics was used to extract radiomics features. A multivariate model was developed using a logistic regression approach. In the training cohort, 85/107 radiomics features were significantly different between patients with benign and malignant IPMNs. Unsupervised clustering analysis revealed four distinct clusters of patients with similar radiomics features patterns with malignancy as the most significant association. The multivariate model differentiated benign from malignant tumors in TC with an area under the ROC curve (AUC) of 0.84, sensitivity (Se) of 0.82, specificity (Spe) of 0.74, and in EVC with an AUC of 0.71, Se of 0.69, Spe of 0.57. This large study confirms the high diagnostic performance of preoperative CT-based radiomics analysis to differentiate between benign from malignant IPMNs.

## 1. Introduction

Intraductal papillary mucinous neoplasms (IPMN) of the pancreas are mucin-producing tumors that originate from the pancreatic ducts. Their incidence keeps growing due to improvement and widespread use of cross-sectional imaging coupled with the population’s increasing age. They account for nearly half of the pancreatic cysts discovered in up to 2.6% of computed tomography (CT) scans and 20% of magnetic resonance imaging (MRI) studies each year [1,2]. Most of the time, they are discovered incidentally for conditions unrelated to the pancreas.

IPMNs comprise a clinically challenging entity since they exhibit a potential risk of invasive transformation and are a precursor of concomitant pancreatic ductal adenocarcinoma (PDAC) in 4–11% of cases [3,4,5,6]. IPMNs can be classified into three types according to the ductal involvement: MD (main duct)-IPMN, BD (branch-duct)-IPMN, and mixed type as well as into four pathologic subtypes: gastric, intestinal, pancreatobiliary, and oncocytic, with each displaying distinct biological behavior [7]. Moreover, the spectrum of architectural and histological dysplasia is a determinant concept for the risk of malignant transformation. Based on the WHO 2019 classification, there are low-grade dysplasia (LGD), high-grade dysplasia (HGD), and invasive intraductal papillary mucinous carcinomas (IPMC) [8]. An aggressive surgical resection approach was initially recommended for the prevention or early treatment of pancreatic cancer. However, this has led to overtreatment, as highlighted in a systematic review of 37 case series that showed that the rate of malignancy development during follow-up is low, with only 2.8% of patients developing invasive neoplasia [9]. In addition, pancreatectomy is responsible for one of the highest rates of morbidity (40–50%) and mortality (2–4%) in abdominal surgery with postoperative complications that include mainly endocrine and exocrine pancreatic insufficiencies as well as the development of steatosis [10,11,12]. Therefore, the current management of IPMN requires the maintenance of a balance between the risk of potential malignant transformation and the risk of pancreatic resection. International guidelines have illustrated this principle with the emergence of a more conservative approach over the last few years in patients at low risk of developing invasive carcinoma or HGD [13,14,15].

IPMNs with obvious “high-risk stigmata” (HRS) on CT/MRI (jaundice, the presence of an enhancing mural nodule (≥5 mm) or a solid component, or a MPD measuring ≥10 mm) are highly predictive of malignancy and should undergo resection in surgically fit patients [13,15].

“Worrisome features” (WF) include cysts of a diameter ≥30 mm, enhancing mural nodule <5 mm, thickened enhanced cyst walls, MPD size of 5–9 mm, an abrupt change in the MPD caliber with distal pancreatic atrophy, lymphadenopathy, an elevated serum level of carbohydrate antigen (CA)19-9, and a rate of cyst growth > 5 mm/2 years. These are also associated with an increased risk of high-grade dysplasia or invasive cancer and can be considered relative indications for surgical resection [13,15] but recommendations differ according to guidelines.

Despite these improvements, the most recent International Consensus Guidelines (ICG) continue to contribute to unnecessary pancreatic resections because morphologic imaging features on CT and/or MRI are still inaccurate for assessing dysplasia [14,16,17]. A multi-institutional study by Wilson et al., which included 324 patients, found that only 44% of the specimens resected had LGD [18]. Lekkerkerker et al. compared the ICGs (2012) [14] with the European (2013) [19] and American guidelines (2015) [20] to retrospectively establish their performance on 75 patients with IPMN. The indications for surgery were then justified in 54%, 53%, and 59% of the patients, respectively [21].

Thus, there is a need to develop new noninvasive biomarkers that could better assess malignancy risk in IPMNs. Radiomics has introduced a new way to mine information contained in medical images. Known as an increasingly common tool in modern radiology practice, it has the ambition to find associations between qualitative and quantitative information [22]. It involves the process of extracting high-dimensional quantifiable features that might correlate with the underlying biology or clinical outcomes using advanced machine learning analysis techniques extracted from clinical images and clinical data. To overcome the wealth of complex parameters, these derived values are implemented into mathematical algorithms to predict a certain clinical outcome as a critical output (e.g., stratifying the risk of malignancy in IPMN). In many oncological applications, significant progress has been made regarding radiomics, serving as biomarkers to improve noninvasive tumor characterization, association with tumor spread, prediction of prognosis, and therapeutic outcomes [23,24,25]. Very few preliminary studies have been published on IPMNs, and their results seem promising but have only analyzed a small patient population [26,27,28,29,30,31,32].

Therefore, this study aimed to investigate a contrast-enhanced CT-based radiomics approach to differentiate between LGD, HGD, and invasive IPMN in a large retrospective series of consecutive patients who underwent pancreatic resection for IPMN.

## 2. Materials and Methods

### 2.1. Patients

Ethical approval was obtained from the Human Research Ethics Committee (HREC) of our hospital. The HREC waived the patient informed consent for the retrospective usage of patients’ medical images.

From January 2005 to December 2018, 595 patients underwent surgical resection at the Department of Pancreatic Surgery, Beaujon Hospital (Clichy, France) with pathologically proven IPMN lesions.

The inclusion criteria were as follows: (i) patients who have undergone pancreatic surgery for IPMN (main duct, side-branch, mixed-type) with a histologically confirmed diagnosis of IPMN; (ii) contrast-enhanced (CE) CT performed within six months from surgery, with pancreatic phase and/or portal venous phase acquisitions.

The exclusion criteria were: (i) patients without a CECT performed within six months before surgery; (ii) inadequate CT technique, i.e., absence of contrast-enhanced CT acquisition, CECT with a reconstruction slice thickness greater than 2.5 mm on pancreatic or portal venous phase; (iii) image non-conformities for image segmentation (e.g., corrupted CT images, segmentation mask with different dimensions than images).

Patients and tumor characteristics were collected from medical charts. Clinical and imaging features included patient age, gender, surgical indications, available CECT phase, time delay between CECT and surgical resection, type of surgery, surgery date, and indication for surgery. Indication for resection was collected according to criteria retained in the ICG and in the recent European recommendations [13,15] as well as other any causes that led to surgical resection.

### 2.2. Training Cohort and External Validation Test Cohort

This study is comprised of two independent datasets. The training cohort aimed to develop the predictive model in 296 patients with CECT performed in our center (Beaujon Hospital, Clichy, France). This cohort consisted of 136 benign IPMNs and 160 malignant IPMNs.

The independent validation cohort was constructed to test the performance of our predictive model. It comprised 112 patients who underwent a CECT performed in various centers in France (*n* = 50) with 45 benign IPMNs and 67 malignant IPMNs.

**Figure 1:**
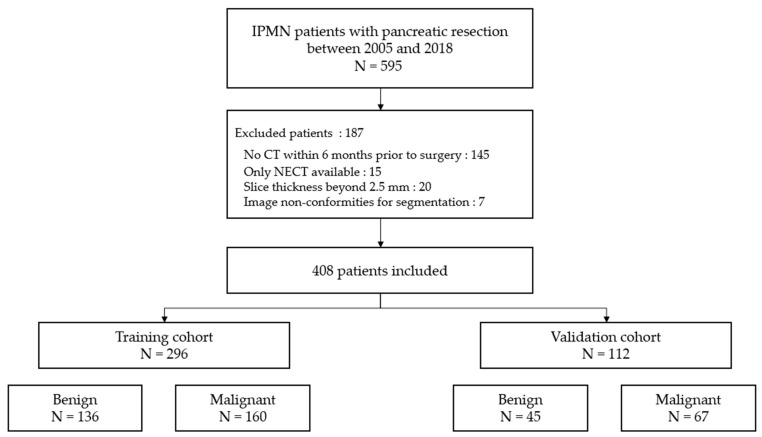
Flow chart of the patient-recruitment process. (NECT): non-enhanced CT.

Both groups fulfilled the same inclusion and non-inclusion criteria. All CT scans were archived within the Picture Archiving and Communication System (PACS, Carestream Health, Rochester, NY, USA) in our hospital. The flow chart is provided in Figure 1.

### 2.3. Surgical Indications

Since January 2005, all clinical, radiological, and biological data of patients undergoing a pancreatectomy in our institution were collected in our archive. We extracted all the features responsible for surgical indications according to the criteria retained in the ICG and the recent European recommendations [13,15] as well as other causes that led to surgical resection.

### 2.4. CECT Protocols

In our institution, contrast-enhanced abdominal CT exams were performed on 64 section multidetector CT scanners (222 patients with LightSpeed VCT, (GE Healthcare, Waukesha, WI, USA) and 62 patients on Discovery CT750 HD LightSpeed (GE Healthcare, Waukesha, WI, USA) using the parameters detailed in Table 1. Contrast-enhanced acquisitions were obtained following intravenous administration into the antecubital vein of 2 mL/kg of non-ionic contrast medium at 350 mg non-ionic/mL with an automatic power injector rate 3 mL/s through an 18- or 20-gauge intravenous catheter.

CECT performed in other institutions were acquired in more than fifty different centers using various CT units. The acquisition parameters matched our inclusion criteria.

### 2.5. Histopathological Data

All cases were handled by expert pancreatic pathologists (A.C, J.C) with, for most cases, a frozen examination of the pancreatic margin to assess whether an extension of the surgery was required. Specimens were then submitted to a standardized macroscopic protocol. Before formol fixation, the main pancreatic duct was inked, and the specimen sliced perpendicularly to the duodenum or the main duct for Whipple and left resection, respectively. This allowed us to precisely map the localization of the cysts and assess the macroscopic main duct involvement. After fixation, the slices were all photographed, and all the cysts were fully sampled for microscopic examination. IPMN lesions were reviewed for this study to classify them according to the digestive WHO 2019 classification (low grade vs. high-grade dysplasia) [33], microscopic IPMN phenotypes, and lymph node metastasis status were collected.

### 2.6. Segmentation

**Figure 2:**
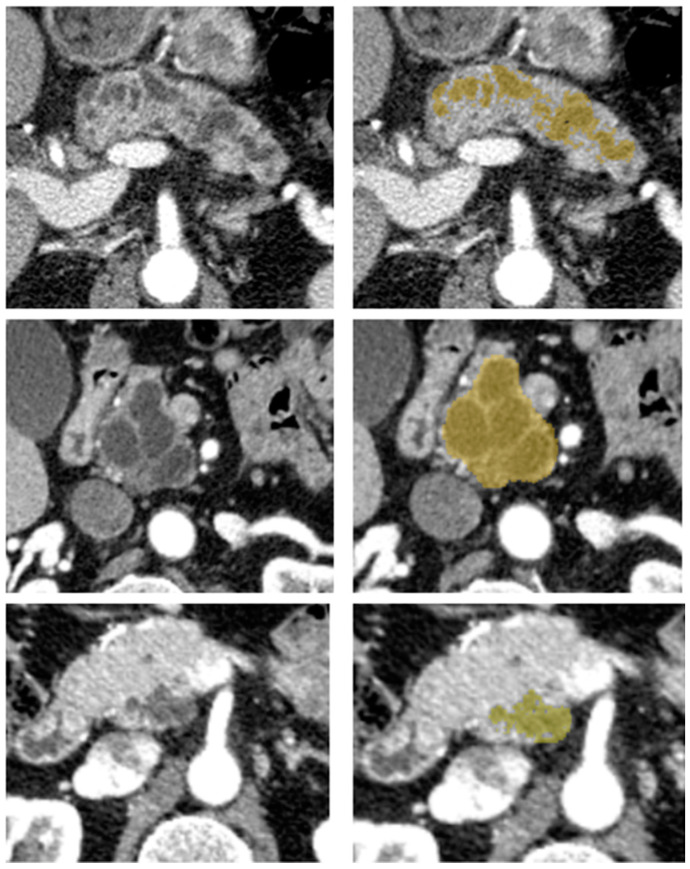
Examples of different IPMN on axial contrast-enhanced CT scans in the pancreatic phase (**left**) and with a semi-automatic outlined tumor in yellow (**right**).

Image segmentation was performed by one reader with five years of abdominal imaging experience. Before segmenting the cohort’s tumors, consensus segmentation was performed on 15 exams, including challenging cases with a senior radiologist who had more than 20 years of experience in IPMN diagnosis. The reader was blinded to the pathologic analysis but not blinded to the type of surgery. Using a web-based medical segmentation tool (MedSeg [34]), delineation of the volume of interest (VOI) was drawn in a semi-automatic manner, covering the tumor’s volume as represented in Figure 2. The semi-automatic segmentation consisted of a smart brush with a variable radius and adjustable minimum and maximum intensity thresholds, preventing voxels with an intensity outside these user-defined thresholds of being segmented, reducing, this way, the number of necessary corrections when compared with a non-thresholded brush segmentation. If multiple cysts were present with no clear evidence of worrisome features, the most conspicuous or larger cyst was selected in the pancreas portion according to the type of surgery (duodenopancreatectomy, left pancreatectomy, enucleation, others). The reader was given no specific directions regarding the display settings such as thresholding and window level. The reader visually inspected the semi-automated segmentation and made free hand corrections when appropriate to reproduce the tumor’s closest shape and avoid including adjacent fat and vessels.

The pancreatic phase was favored for tumor segmentation. When it was not available, a portal venous phase was used for image segmentation and analysis [35,36,37,38].

### 2.7. Radiomics Feature Extraction Methodology

PyRadiomics [39] version 2.2.0 [40] was used to extract 107 radiomics features from the CT-examinations and corresponding tumor segmentation masks. Shape, first-order, Gray Level Co-occurrence Matrix (GLCM), Grey-Level Run Length Matrix (GLRLM), Gray Level Size Zone Matrix (GLSZM), Neighboring Gray Tone Difference Matrix (NGTDM), and Gray Level Dependence Matrix (GLDM) features were extracted. Due to the quantitative nature of CT images [41,42], a fixed bin size was optimized to ensure that the range of the intensity of lesions would be from 30 to 130 bins across patients. An optimal bin size of 3 Hounsfield units was selected to discretize image intensities, part of the extraction of non-shape features. Due to the heterogeneity of the through-plane resolution within the overall dataset, a 2D feature extraction scheme was used where only the in-plane resolution was standardized to 1.00 mm × 1.00 mm across images using a B-Spline interpolation. Resegmentation using an intensity outlier filtering was applied. In this intensity outlier filtering, the mean and standard deviation of each volume of interest was determined, and voxels were discarded if they lay outside of the range mean ± 3 standard deviations.

### 2.8. Statistical Analysis and Machine Learning Modeling

#### 2.8.1. Univariate Analysis

Univariate analysis was conducted to assess each radiomic feature’s power to significantly discriminate between benign and malignant IPMN and between different degrees of dysplasia in the training cohort. The Shapiro–Wilk test was used to assess the normality of the distribution of radiomics features in each group. The *t*-test was applied to assess whether both groups were statistically different based on their radiomic feature values when the distribution of each group was normal. In cases where the feature value distribution of one or both groups was not normally distributed, the Mann–Whitney U test was used to assess group differences. The statistical significance level, α, was set at 0.05, and the Holm method (uniformly more powerful than the Bonferroni method) was used to reduce Type I errors because of the multiple comparisons. The classification performance of features was assessed using the area under the Receiver Operating Characteristic (ROC) curve, and the Youden index was used to obtain the optimal cut-off of separation between groups, and corresponding accuracy, sensitivity (Se), and specificity (Spe) on the training cohort. The optimal cut-off for each feature determined using the training cohort was then applied to the external validation test cohort and accuracy, Se, and Spe was determined.

#### 2.8.2. Unsupervised Clustering

Unsupervised clustering was used to investigate possible associations between groups of radiomics features with similar characteristics and clinical, biological, or radiological variables leading to surgical resection. The Euclidean distance was used as the clustering distance metric across features and patients, and the agglomerative hierarchical clustering method chosen was the average linkage. The chi-square test was used to test associations between patient clusters and clinical parameters.

#### 2.8.3. Multivariate Modeling

The model development and tuning consisted of several steps. Initially, the radiomics features with near-zero and zero variance on the training dataset were identified and removed. Consequently, highly correlated features were removed using a correlation coefficient threshold of 0.95. The remaining features were used to develop and tune the models.

**Figure 3:**
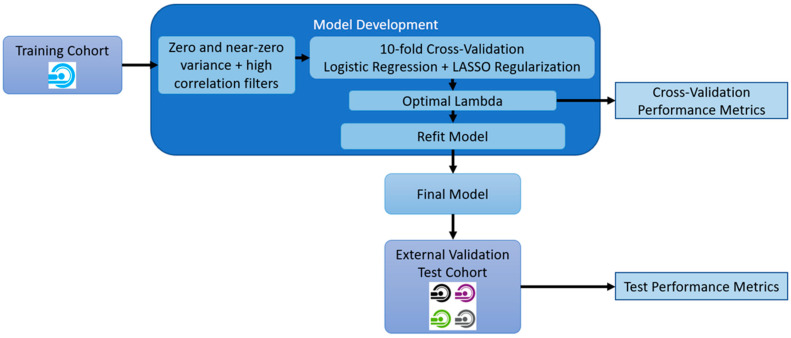
Overview of model development and model assessment procedures.

Two pairs of models to classify IPMNs were designed. The first used radiomics features only (referred to as the radiomics model). In the second model, radiomics features were associated with the surgical indication variables known to guide the patient to surgery (referred to as radiomics + surgical indication variables). These analyses were developed and assessed for several scenarios: benign versus malignant IPMNs and between different degrees of dysplasia. For the development of these models, Logistic Regressions with Least Absolute Shrinkage and Selection Operator (LASSO) regularizations, which is used to perform high-dimensional data regression analysis, penalizing model complexity and selecting the most informative features, were tuned using the training dataset. A ten-fold cross-validation tuning procedure with the area under the Receiver Operating Characteristic (ROC) Curve (AUC) as the optimization metric was used to determine the optimal regularization parameter lambda. Additionally, the accuracy, Se, Spe, Positive Predictive Value (PPV), Negative Predictive Value (NPV), and Matthews Correlation Coefficients (MCC) were also calculated. The overview of the model development is illustrated in Figure 3.

The different scenarios were also assessed only on the subgroup of branch duct IPMN patients. Due to the higher-class imbalances, the MCC was used as the optimization metric, and the models were developed using a Naïve Bayes classifier.

The trained models were then applied to the external validation cohort to predict the corresponding outcomes, and AUC, accuracy, Se, Spe, PPV, NPV, and MCC were calculated. The 95% Confidence Interval (CI) values for each performance metric, except the AUC, were obtained assuming the binomial distribution’s approximation to a Gaussian, which allows the proportion of Gaussian distribution to estimate the confidence intervals. As for the AUC, the CI was obtained using the DeLong method.

Univariate analysis, unsupervised clustering, multivariate modeling, and statistical analysis were performed using caret, stats, pROC, ggplot2, ggpubr, pheatmap, and dendextend packages in R Studio (version 1.1.383–R version 3.5.3) [43,44,45,46,47,48,49,50].

## 3. Results

### 3.1. Study Population

From January 2005 to December 2018, 408 patients with pathologically diagnosed IPMN were eligible in this retrospective study. The training cohort consisted of 152 males and 144 females (range 30–82 years and mean 63.5 years), and the validation cohort included 58 males and 54 females (range 36–84 years and mean 63.2 years). There were no statistically significant differences in category, age, gender, clinical features (jaundice, acute pancreatitis), laboratory tests, type of surgery, anatomical classification, the grade of dysplasia, phenotype classification, and lymphadenopathy on specimens between the training and validation cohorts (*p* > 0.05). The differences in WF/HRS (*p* = 0.008), available CECT phases (*p* = 2.0 × 10^−6^), and days between CECT and surgical resection (*p* = 6.0 × 10^−8^) were statistically significant between the training cohort and the validation cohort. More details are shown in patient characteristics reported in Table 2.

A sub-group analysis of patients with BD-IPMN was individualized. Further details are available in Table S1.

### 3.2. Univariate Analysis

The univariate analysis performed on the training cohort showed a total of 85 (79%) radiomics features with significant differences between patients with benign and malignant IPMNs. A subset of significant features with an AUC > 0.725 is shown in Table 3 (the full list is provided in the Supplementary Material Table S2). The optimal cut-off was used to assess the accuracy, sensitivity, and specificity on the external validation cohort.

### 3.3. Unsupervised Clustering

**Figure 4:**
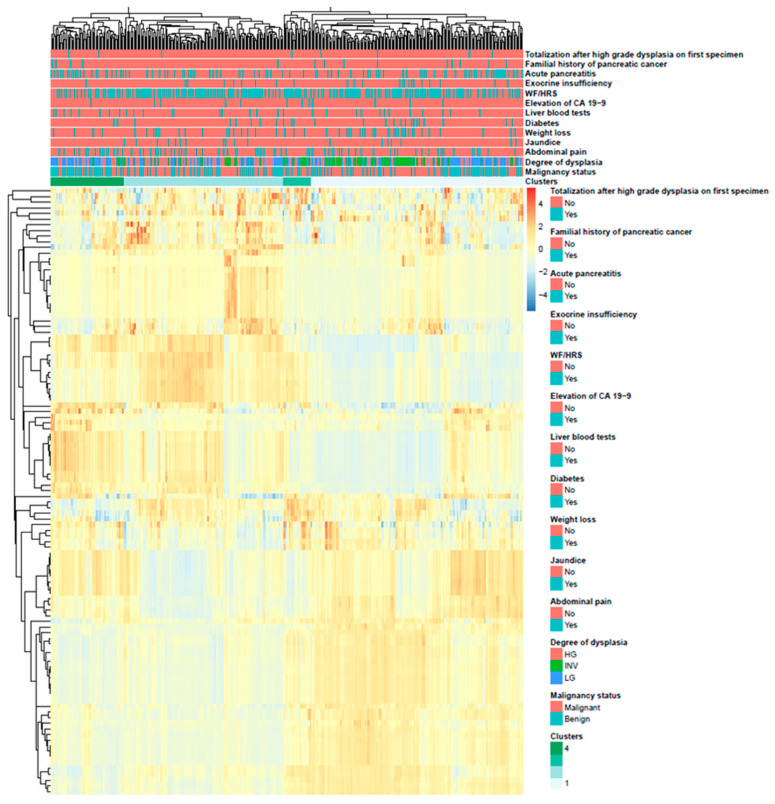
Radiomics heatmap with unsupervised clustering. Four distinct clusters of patients with similar radiomic feature patterns and corresponding malignancy status, degrees of dysplasia, and clinical variables indicating surgery are provided on top of the heatmap.

Unsupervised clustering enabled the identification of four distinct clusters of patients with similar radiomic feature patterns, and the comparison between these clusters and corresponding malignancy status, degrees of dysplasia, and clinical variables providing an indication to surgery was performed and presented in Figure 4. Statistically significant associations with the obtained clusters were found for malignancy status (*p* = 0.002), jaundice (*p* = 0.039), elevation of CA 19-9 (*p* = 0.038), WF/HRS (*p* = 0.024), acute pancreatitis (*p* = 0.015), and familial history of pancreatic cancer (*p* = 0.040). The degree of dysplasia (*p* = 1.000) did not show significant associations with patients’ obtained clusters.

### 3.4. Multivariate Analysis

#### 3.4.1. Whole Population

**Figure 5:**
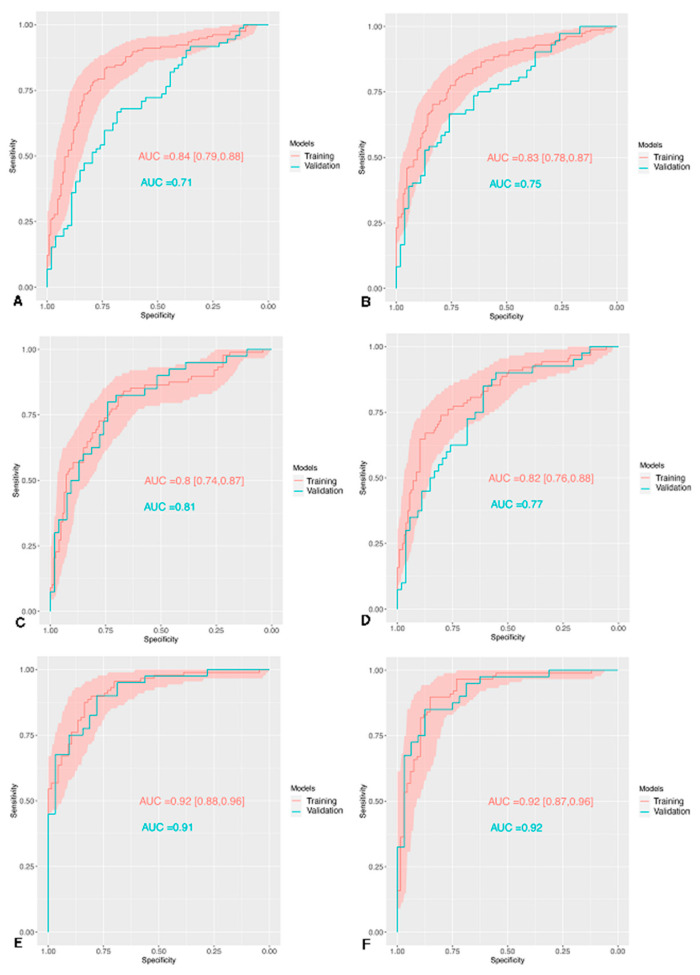
Receiver operating characteristic (ROC) curves for the training and external validation cohorts, in red and green respectively, with radiomics-only (**A**,**C**,**E**) and radiomics + surgical indication variables (**B**,**D**,**F**) models in red and green respectively through different scenarios: (**A**,**B**) Benign vs. Malignant IPMN, (**C**,**D**) low grade dysplasia vs. high grade dysplasia, (**E**,**F**) high grade dysplasia vs invasive intraductal papillary and mucinous neoplasm of the pancreas. (AUC): Area Under the ROC curve.

The multivariate model of radiomics-only differentiated benign from malignant tumors in the training cohort with an AUC of 0.84 (95% CI: 0.79–0.88), Se of 0.82 (95% CI: 0.81–0.83), Spe of 0.74 (95% CI: 0.71–0.77), and in the external validation cohort with an AUC of 0.71, Se of 0.69, Spe of 0.57. The performance of the radiomics + surgical indication variables model demonstrated an AUC of 0.83 (95% CI: 0.78–0.87), Se of 0.80 (95% CI: 0.79–0.81), Spe of 0.72 (95% CI: 0.69–0.74), and in the external validation cohort with an AUC of 0.75, Se of 0.69, Spe of 0.65. Further parameters are detailed in Table 4 and represented in the Figure 5A,B.

The results of similar models developed for the discrimination of the subgroup of patients with low-grade and high-grade dysplasia presented in the training cohort with an AUC of 0.80 (95% CI: 0.74–0.87), Se of 0.72 (95% CI: 0.68–0.75), Spe of 0.78 (95% CI: 0.75–0.81), and in the external validation cohort with an AUC of 0.81, Se of 0.68, Spe of 0.76. The performance of the radiomics + surgical indication variables model demonstrated an AUC of 0.82 (95% CI: 0.76–0.88), Se of 0.74 (95% CI: 0.69–0.78), Spe of 0.79 (95% CI: 0.76–0.81), and in the external validation cohort with an AUC of 0.77, Se of 0.62, Spe of 0.70. Further parameters are detailed in Table S3 and Figure 5C,D.

Similarly, the 3-class models tuned for the discrimination between the three different degrees of dysplasia were also assessed, and their performances on the cross-validation and external validation. In the training cohort, there was an AUC of 0.81, Se of 0.72 (95% CI: 0.69–0.75), Spe of 0.86 (95% CI: 0.84–0.87), and in the external validation cohort with an AUC of 0.73, Se of 0.67, 0.63 and 0.53 and Spe of 0.67, 0.86 and 0.87 for LGD, HGD and invasive respectively. The performance of the radiomics + surgical indication variables model demonstrated an AUC of 0.81, Se of 0.74 (95% CI: 0.73–0.76), Spe of 0.87 (95% CI: 0.86–0.87), and in the external validation cohort with an AUC of 0.73, Se of 0.63, 0.65 and 0.59 and Spe of 0.71, 0.83 and 0.88 for LGD, HGD and invasive, respectively. Further parameters are detailed in Table S4 and Figure 5E,F.

Two models for the differentiation between high-grade and invasive pancreatic IPMNs using only radiomics features and radiomics features with surgical indication variables were also developed and evaluated. The training cohort had an AUC of 0.92 (95% CI: 0.88–0.96), Se of 0.81 (95% CI: 0.76–0.85), Spe of 0.89 (95% CI: 0.86–0.91), and in the external validation cohort with an AUC of 0.91, Se of 0.69, Spe of 0.95. The performance of the radiomics + surgical indication variables model demonstrated an AUC of 0.92 (95% CI: 0.87–0.96), Se of 0.82 (95% CI: 0.78–0.86), Spe of 0.90 (95% CI: 0.88–0.92), and in the external validation cohort with an AUC of 0.92, Se of 0.69, Spe of 0.95. Further parameters are detailed in Table S5.

All the ROC curves for the training and external validation cohorts for the differentiation between benign and malignant, low-grade and high-grade, and high-grade and invasive IPMN in the whole population are provided in Figure 5.

The models’ coefficients for the different scenarios are shown in Figure S1.

#### 3.4.2. Subgroup of BD-IPMNs

Additionally, models for the subgroup of patients with branch duct (BD) IPMNs were developed, and corresponding performances were calculated. The performance of the radiomics and radiomics with surgical indication variable models developed for the discrimination between benign and malignant BD-IPMN. The results showed in the training cohort an AUC of 0.73 (95% CI: 0.62–0.83), Se of 0.65 (95% CI: 0.57–0.73), Spe of 0.69 (95% CI: 0.62–0.76), and in the external validation cohort with an AUC of 0.55, Se of 0.36, Spe of 0.72. The performance of the radiomics + surgical indication variables model demonstrated an AUC of 0.73 (95% CI: 0.63–0.84), Se of 0.72 (95% CI: 0.64–0.79), Spe of 0.63 (95% CI: 0.57–0.69), and in the external validation cohort with an AUC of 0.57, Se of 0.50, Spe of 0.64. Further parameters are detailed in Table 5.

Similarly to the whole population analysis, we developed models in the BD-IPMNs, using only radiomics features and radiomics features + surgical indication variables to discriminate between LGD and HGD, between HGD and invasive, as well as LGD vs. HGD vs. invasive (Tables S6–S8).

## 4. Discussion

In this study, we have shown in a large cohort of IPMN patients that radiomics enable distinction of the different IPMN grades, particularly benign (low-grade dysplasia) from malignant (high-grade dysplasia and invasive carcinoma) ones. As it is a very challenging issue with current clinicobiological and radiological data, we think it could contribute to better patient management.

Eighty-five radiomics features showed the statistical power to discriminate between benign and malignant IPMNs and the top ten features were first- or second-order radiomics features with AUCs above 0.725 distinguishing benign from malignant. A common strategy to explore if useful patterns or otherwise called “machine learning signal”, are present in our data, is to perform unsupervised clustering analysis In our study, it revealed four distinct clusters of patients with similar radiomic features patterns. Among the significant associations, malignancy was the most significant, providing evidence that the computed radiomic phenotypes are significantly different between benign and malignant IPMNs. The next step is multivariate modeling where there is a need to identify the subset of features otherwise called radiomics signatures, that can be used to predict the final outcome with high performance and robustness.

In our study, the multivariate model used only radiomic features had high AUC in the training and in the external validation cohorts (0.84 and 0.71, respectively). This is the first large radiomics study using a highly diverse multiple external validation cohort. Preliminary radiomic studies in IPMN [26,27,32] (including 38, 51, and 53 patients) have shown similar diagnostic performance for differentiating benign from malignant IPMN with AUC values ranging between 0.77 and 0.87.

These diagnostic performances challenge the classical criteria used to differentiate benign from malignant IPMN including a combination of clinicobiological and radiological criteria, the most common being worrisome features and high-risk stigmata or imaging findings alone. In a meta-analysis comprising 21 studies with 3723 patients with cystic lesions of the pancreas (mostly IPMN), the 2012 Fukuoka and the AGA guidelines had an AUC of 0.78. and 0.79, for the prediction of malignancy, respectively [51]. Comparison of the 2012 and 2017 Fukuoka guidelines even showed a decrease in specificity translating into futile pancreatic resection and stressing the fact that there is a need to improve IPMN classification [16]. Using CT or MRI features only, the presence of an enhancing mural nodule, main pancreatic duct size, abrupt main pancreatic duct caliber change, and lymphadenopathy were more common in malignant than benign IPMNs resulting in AUCs of 0.83 and 0.86 with CT and MRI, respectively [52]. Besides recommendations, nomograms have been tested and validated in Eastern and Western patients, with AUC for combined cohorts in the range of previous works (0.776) [53].

Because BD-IPMN are less prone to malignancy, and only 18% of resected BD-IPMN were malignant at pathology, distinction between benign and malignant is even more important in BD-IPMN than in other types [54], justifying the conduction of radiomics analyses in this subgroup. AUCs were slightly below the results obtained in the whole population (0.73 and 0.55 in the training and in the external validation cohort, respectively). Yet, they are consistent with those reported by preliminary radiomics studies [30,31]. Similarly to IPMNs as a whole, diagnostic performance of imaging criteria has been shown to be low in a meta-analysis focusing on BD-IPMN [55]. The best imaging features was the presence of a mural nodule with an AUC 0.786, a pooled sensitivity of 59% and a pooled specificity of 83% [55].

Whatever the approach (clinical, radiological, or quantitative), sensitivities and specificities do not reach 90% and therefore do not adequately stratify and identify patients at risk of having high-risk dysplasia or invasive cancer.

In our study, we have also evaluated the ability of combined qualitative (surgical indications) and quantitative data to discriminate benign from malignant IPMN. We obtained diagnostic performances similar to the radiomics-only approach (AUC of 0.83 and 0.75) in the training and in the external validation cohort. Lack of major improvement was also observed in preliminary series and could be explained by the fact that surgical indication variables have highly selected the patient population that should be resected [30]. Only one study reported a significant increase in AUC by combining CT radiomics features with matched plasma-based miRNA genomic classifier in 38 patients [27].

We also developed similar models for the discrimination of patients’ subgroups: low-grade vs. high-grade dysplasia, high-grade dysplasia vs. invasive carcinoma, and low-grade vs. high-grade dysplasia vs. invasive carcinoma. AUCs ranged from 0.80–0.92 in the training cohort and 0.73–0.91 in the external validation cohort. Interestingly, the highest diagnostic performance was observed in distinguishing high-grade dysplasia and invasive pancreatic IPMNs. This may have an important clinical impact as increasing data have shown the benefits of neoadjuvant chemotherapy in pancreatic adenocarcinoma, explaining the shift from upfront surgery to multidisciplinary treatment of even resectable pancreatic cancer [56].

Due to the relatively high number of radiomic features, there is an increased likelihood of overfitting. In the case of the IPMN patients, Logistic Regression models were regularized using LASSO to ensure that not all features were used in the models, as this method penalizes the model complexity and selects the most informative features. As for the Naïve Bayes models used for BD-IPMNs, no regularization was utilized, which could explain the difference in performance between the training and external testing cohorts.

Conversely to the preliminary radiomics studies in IPMN, ours comprised a large training cohort and an external validation cohort. Interestingly, our training cohort was composed of patients examined at our institution with a highly standardized acquisition protocol and few CT units, while the external validation cohort were patients who had their CT examinations performed outside our institution with various CT machines and protocols (including timing of the contrast-enhanced phases). Overall, the general performance of the algorithms was not altered in the external validation cohort, demonstrating the ability of the study to allow for generalization of the study results to the target population [57].

MRI is the modality of choice in IPMN as it is far more sensitive than CT in detecting cystic lesions of the pancreas, probably due to the higher contrast resolution of MRI, and for identifying communication between a pancreatic cystic neoplasm and the pancreatic duct system [13]. However, we have chosen to evaluate CT radiomics in patients with IPMN. Noticeably, pancreatic CT and pancreatic MRI/MRCP have a similar accuracy for the characterization of pancreatic cystic neoplasms [58,59]. In IPMNs, diagnostic performances of contrast-enhanced CT and MRI for prediction of malignancy are similar without demonstrated significant differences [60,61]. Moreover, MRI would have been much more difficult because it suffers from less standardization than CT with a large variety in the multiple parameters related to scanner properties, acquisition settings, and image processing that could hamper radiomics analysis. However, further studies should also consider MR-based radiomics.

Another interesting topic would be to analyze CT- or MR-based radiomics in patients who had serial imaging examinations in order to evaluate the potential of radiomics in assessing changes that would not be captured by morphological criteria.

Our study has limitations. First, this is a retrospective unicentric study, which could reduce generalization of the results. Yet, our patient population exceeds 400 patients and we have set up an external validation cohort reflecting the daily practice. As the method of reference is the pathologic examination of the resected pancreas, we also wanted to benefit from our expert pathologists. Second, the segmentation was semi-automatic and performed by one radiologist. Segmentation is indeed a critical step of the radiomics process because data are extracted from the segmented volumes. Fully automatic segmentation methods have not been used in this study because they were very challenging in poorly defined IPMNs. Where manual segmentation can suffer from inter-observer variability, it has been shown that semi-automated approaches are fast and reduce inter-observer variability [62]. Moreover, the first 15 IPMN cases have been segmented by the radiologist and reviewed by an expert radiologist in pancreatic diseases.

## 5. Conclusions

In conclusion, our large study shows high diagnostic performance of CT radiomics in differentiating benign from malignant IPMNs as well as in subgroups classification. Results obtained from the training and external validation cohorts were similar, enabling generalization of the proposed models. There is still room for improvement and further large studies should perform head-to head comparisons of the performance of radiomics-based and imaging-based approaches, as well as in combination.

## Figures and Tables

**Table 1:** Details of CT protocols in our institution for both CT scans.

	Study Cohort	
	Discovery CT750 HD Lightspeed	Discovery Lightspeed VCT
Number of patients	62	222
Number of channels	64	64
Tube current	Modulated tube current	Modulated tube current
Tube voltage	120 kV	120 kV
Gantry rotation time	0.5 s	0.5 s
Helical pitch	0.60–1.10	0.60–1.10
Reconstruction algorithm	FBP (Filtered Back Projection)	FBP (Filtered Back Projection)
Slice thickness	0.625–2.5 mm	0.625–2.5 mm
Slice interval	1.5–2 mm	1.5–2 mm
Matrix size	512 × 512	512 × 512
Kernel	B20f	B20f
**Pancreatic phase**		
Pitch	0.98	0.98
Delay after injection	45 s	45 s
**Portal phase**		
Pitch	0.98	0.98
Delay after injection	80 s	80 s
Contrast agent	350 mg/mL (non-ionic)	350 mg/mL (non-ionic)
Volume	2 mL/kg	2 mL/kg
Rate	3 mL/s	3 mL/s

**Table 2:** Detailed patients’ characteristics in the training and external validation cohorts.

	Training Cohort (296)		External Validation Cohort (112)		*p*
**Category**	Benign (136) (46%)	Malignant (160) (54%)	Benign (45) (40%)	Malignant (67) (60%)	0.30
**Age (mean, range)**	61.8 (30–81)	65.2 (41–82)	60.5 (38–77)	66.0 (36–84)	0.47
**Gender**					0.79
Male	57 (42%)	95 (59%)	23 (51%)	35 (52%)	
Female	79 (58%)	65 (41%)	22 (49%)	32 (48%)	
**Surgical indications**					
**Clinical features**					
Jaundice	0 (0%)	10 (6%)	1 (2%)	7 (10%)	0.10
Acute pancreatitis	52 (38%)	28 (18%)	14 (31%)	9 (13%)	0.18
**Laboratory tests**					
CA 19.9 ≥ 37 UI/mL	3 (2%)	5 (3%)	0 (0%)	4 (6%)	0.64
**Radiological features**					
WF/HRS	63 (46%)	103 (64%)	26 (58%)	53 (79%)	0.008
**Others ^1^**	73 (54%)	101 (63%)	30 (67%)	41 (61%)	0.39
**CECT phase**					2.0 × 10^−6^
Pancreatic phase	118 (87%)	144 (90%)	33 (73%)	44 (66%)	
Portal phase	18 (13%)	16 (10%)	12 (27%)	23 (34%)	
**Days between CECT and surgical resection (mean, range)**	57.4 (1–180)	51.0 (1–163)	98.3 (5–180)	72.8 (12–161)	6.0 × 10^−8^
**Type of surgery**					0.09
Duodeno-pancreatectomy	62 (46%)	98 (61%)	15 (33%)	38 (57%)	
Left pancreatectomy	30 (22%)	38 (24%)	13 (29%)	22 (33%)	
Other	44 (32%)	24 (15%)	17 (38%)	7 (10%)	
**Anatomical classification**					0.52
Main-Duct IPMN	3 (2%)	9 (6%)	1 (2%)	3 (4%)	
Branch-Duct IPMN	73 (54%)	31 (19%)	19 (42%)	14 (21%)	
Mixed-Type IPMN	60 (44%)	120 (75%)	25 (56%)	50 (75%)	
**Grade dysplasia**	Low grade (136)	High grade (92) Invasive (68)	Low grade (45)	High grade (36) Invasive (31)	0.58
**Phenotype classification (from 2012)**					0.50
Gastric	44 (32%)	37 (23%)	16 (36%)	19 (28%)	
Intestinal	25 (18%)	50 (31%)	6 (13%)	22 (33%)	
Pancreatobiliary	1 (1%)	10 (6%)	1 (2%)	3 (4%)	
Oncocytic	0	1 (1%)	0 (0%)	2 (3%)	
**Lymphadenopathy on specimen**	0	29 (18%)	0 (0%)	10 (15%)	0.79

^1^ Abdominal pain, weight loss, diabetes, exocrine insufficiency, familial history of pancreatic cancer, totalization after high-grade dysplasia on the first specimen, abnormal blood liver tests. IPMN: Intraductal papillary mucinous neoplasms; CA 19.9: cancer antigen 19.9; CECT: contrast-enhanced CT; WF: worrisome findings; HRS: high risk stigmata.

**Table 3:** Subset of radiomics features in the training cohort and the external validation cohort presenting statistically significant differences between benign and malignant groups. The adjusted *p*-value, AUC, optimal cut-off, accuracy, sensitivity, and specificity are provided for the training cohort.

Features	Training Cohort						External Validation Cohort		
	*p*-Value adj.	AUC	Cut-Off	Accuracy	Sensitivity	Specificity	Accuracy	Sensitivity	Specificity
**First-order** **Energy**	<0.001	0.751	19,284,480.500	0.693	0.652	0.744	0.577	0.479	0.702
**First-order** **Total Energy**	<0.001	0.750	24,105,600.625	0.690	0.646	0.744	0.615	0.466	0.807
**GLDM Small Dependence Low Gray Level Emphasis**	<0.001	0.747	0.002	0.711	0.753	0.659	0.538	0.411	0.702
**GLSZM Small Area Low ** **Gray Level Emphasis**	<0.001	0.742	0.002	0.711	0.785	0.620	0.538	0.438	0.667
**GLRLM Short Run Low Gray Level Emphasis**	<0.001	0.740	0.002	0.700	0.734	0.659	0.500	0.356	0.684
**GLRLM Low Gray Level Run Emphasis**	<0.001	0.739	0.002	0.704	0.734	0.667	0.508	0.370	0.684
**GLSZM Low Gray Level Zone Emphasis**	<0.001	0.739	0.003	0.711	0.804	0.597	0.531	0.438	0.649
**GLDM Low Gray Level Emphasis**	<0.001	0.738	0.002	0.704	0.734	0.667	0.508	0.370	0.684
**GLRLM Long Run Low Gray Level Emphasis**	<0.001	0.733	0.003	0.697	0.766	0.612	0.523	0.425	0.649
**GLCM Idmn**	<0.001	0.730	0.974	0.693	0.696	0.690	0.500	0.562	0.421
**GLCM Idn**	<0.001	0.728	0.891	0.686	0.671	0.705	0.500	0.548	0.439

**Table 4:** Cross-validation and external validation performance of models using only radiomics features and using radiomics features + clinical variables to differentiate between benign and malignant pancreatic IPMN patients.

	Radiomics Only		Radiomics + Surgical Indication Variables	
	CV Mean (95% CI)	External Validation	CV Mean (95% CI)	External Validation
AUC	0.84 (0.79–0.88)	0.71	0.83 (0.78–0.87)	0.75
Acc	0.78 (0.77–0.80)	0.64	0.76 (0.75–0.77)	0.67
Se	0.82 (0.81–0.83)	0.69	0.80 (0.79–0.81)	0.69
Spe	0.74 (0.71–0.77)	0.57	0.72 (0.69–0.74)	0.65
PPV	0.80 (0.78–0.82)	0.68	0.78 (0.77–0.80)	0.72
NPV	0.77 (0.75–0.78)	0.58	0.75 (0.74–0.76)	0.61
MCC	0.56 (0.53–0.59)	0.27	0.52 (0.50–0.54)	0.34

(CV): cross-validation; (AUC): Area Under the receiver operating characteristic Curve; (Acc): accuracy; (Se): sensitivity; (Spe): specificity; (PPV): positive predictive values; (NPV): negative predictive values; (MCC): Matthews Correlation Coefficient.

**Table 5:** Cross-validation and external validation performance of models using only radiomics features and using radiomics features + clinical variables to differentiate between benign and malignant pancreatic BD-IPMN patient.

	Radiomics Only		Radiomics + Surgical Indication Variables	
	CV Mean [(95% CI)	External Validation	CV Mean (95% CI)	External Validation
AUC	0.73 (0.62–0.83)	0.55	0.73 (0.63–0.84)	0.57
Acc	0.68 (0.61–0.74)	0.59	0.65 (0.59–0.71)	0.59
Se	0.65 (0.57–0.73)	0.36	0.72 (0.64–0.79)	0.50
Spe	0.69 (0.62–0.76)	0.72	0.63 (0.57–0.69)	0.64
PPV	0.54 (0.44–0.63)	0.42	0.48 (0.40–0.55)	0.44
NPV	0.81 (0.76–0.85)	0.67	0.83 (0.78–0.88)	0.70
MCC	0.34 (0.21–0.48)	0.08	0.33 (0.20–0.45)	0.14

(CV): cross-validation; (AUC): Area Under the receiver operating characteristic Curve; (Acc): accuracy; (Se): sensitivity; (Spe): specificity; (PPV): positive predictive values; (NPV): negative predictive values; (MCC): Matthews Correlation Coefficient.

## Supplementary Materials

The following are available online at https://www.mdpi.com/2072-6694/12/11/3089/s1, Table S1: BD-IPMN patients characteristics for the training and the external validation cohorts, Table S2: Complementary subset of radiomic features in the training cohort and the external validation cohort presenting statistically significant differences between benign and malignant groups, Table S3: Cross-validation and external validation performance of models using only radiomic features and using radiomic features + clinical variables to differentiate between low-grade and high-grade pancreatic IPMN patients, Table S4: Cross-validation and external validation performance of models using only radiomic features and using radiomic features + clinical variables for the differentiation between patients with low-grade, high-grade, and invasive pancreatic IPMNs, Table S5: Cross-validation and external validation performance of models using only radiomic features and using radiomic features + clinical variables to differentiate between patients with high-grade and invasive pancreatic IPMNs, Table S6: Cross-validation and external validation performance of models using only radiomic features and using radiomic features + clinical variables to differentiate between low-grade and high-grade pancreatic BD-IPMN patients, Table S7: Cross-validation and external validation performance of models using only radiomic features and using radiomic features + clinical variables to differentiate between patients with low-grade, high-grade, and invasive pancreatic IPMNs, Table S8: Cross-validation and external validation performance of models using only radiomic features and using radiomic features + clinical variables to differentiate between patients with high-grade and invasive pancreatic BD-IPMNs, Figure S1: Coefficients of selected variables for (A) radiomics and (B) radiomics + surgical indications models for the differentiation between benign and malignant IPMNs. (C) and (D) provide the coefficients of selected variables for radiomics and radiomics + surgical indications models for the differentiation of low-grade and high-grade dysplasia IPMNs, while (E) and (F) show the coefficients of radiomics and radiomics + surgical indications models for differentiation between high-grade and invasive IPMNs.
