# Supplementary material for: CT-Based Radiomics Analysis to Predict Malignancy in Patients with Intraductal Papillary Mucinous Neoplasm (IPMN) of the Pancreas
Source: Cancers (Basel). 2020 Oct 23;12(11):3089. doi: 10.3390/cancers12113089 (PMC7690711; doi:10.3390/cancers12113089)
Supplement: Supplementary file 1 [file cancers-12-03089-s001.pdf]

cancers MDPI

*Supplementary Materials*

# CT-based radiomics analysis to predict malignancy in patients with intraductal papillary mucinous neoplasm (IPMN) of the pancreas

**David Tobaly [1,*,†], Joao Santinha [2,3,†], Riccardo Sartoris [1,4], Marco Dioguardi Burgio [1,4], Celso Matos [5,6], Jérôme Cros [7], Anne Couvelard [8], Vinciane Rebours [9], Alain Sauvanet [10], Maxime Ronot [1,4], Nikolaos Papanikolaou [11,†] and Valérie Vilgrain [1,4,*,†]**

**Table 1.** Detailed patients' characteristics in the training and validation cohort.

| **Branch Duct-IPMN** | **Training cohort (101)** | | **Testing cohort (32)** | | ***p*** |
|---|---|---|---|---|---|
| **Category** | Benign (72) (71%) | Malignant (29) (29%) | Benign (19) (59%) | Malignant (13) (41%) | 0.22 |
| **Age (mean, range)** | 58.6 (30-82) | 62.8 (42-74) | 58.5 (38-74) | 65.8 (55-75) | 0.20 |
| **Gender** | | | | | 0.41 |
| Male | 32 (44%) | 20 (69%) | 11 (58%) | 8 (62%) | |
| Female | 40 (56%) | 9 (31%) | 8 (42%) | 5 (38%) | |
| **Surgical indications** | | | | | |
| **Clinical features** | | | | | |
| Jaundice | 0 (0%) | 1 (3%) | 1 (5%) | 2 (15%) | 0.02 |
| Acute pancreatitis | 34 (47%) | 9 (31%) | 9 (47%) | 2 (15%) | 0.41 |
| **Laboratory tests** | | | | | |
| Elevation of CA 19-9 | 2 (3%) | 0 (0%) | 0 (0%) | 0 (0%) | 0.42 |
| **Radiological features** | | | | | |
| WF / HRS | 24 (33%) | 19 (66%) | 9 (47%) | 9 (69%) | 0.18 |
| **Others*** | 40 (56%) | 7 (24%) | 23 (121%) | 7 (54%) | $2.4x10^{-6}$ |
| **CECT phase** | | | | | 0.02 |
| Pancreatic phase | 63 (88%) | 27 (93%) | 16 (84%) | 7 (54%) | |
| Portal phase | 9 (13%) | 2 (7%) | 3 (16%) | 6 (46%) | |
| **Days between CECT and surgical resection (mean, range)** | 64.7 (1-180) | 51.3 (1-132) | 96.3 (5-180) | 70.3 (12-139) | 0.01 |
| **Type of surgery** | | | | | 0.86 |
| Duodenopancreatectomy | 32 (44%) | 17 (59%) | 7 (37%) | 8 (62%) | |
| Left pancreatectomy | 10 (14%) | 2 (7%) | 2 (11%) | 3 (23%) | |
| Others | 29 (40%) | 10 (34%) | 12 (63%) | 2 (15%) | |
| **Grade dysplasia** | Low grade (72) | High grade (22) Invasive (7) | Low grade (19) | High grade (7) Invasive (6) | 0.14 |
| **Phenotype classification (from 2012)** | | | | | 0.93 |
| Gastric | 23 (32%) | 6 (21%) | 7 (37%) | 3 (23%) | |
| Intestinal | 7 (10%) | 2 (7%) | 4 (21%) | 0 (0%) | |
| Pancreatobiliary | 1 (1%) | 11 (38%) | 0 (0%) | 4 (31%) | |
| Oncocytic | 0 (0%) | 0 (0%) | 0 (0%) | 0 (0%) | |
| **Lymphadenopathy on specimen** | 0 (0%) | 2 (7%) | 0 (0%) | 1 (8%) | 0.70 |

[1]Abdominal pain, weight loss, diabetes, exocrine insufficiency, familial history of pancreatic cancer, totalization after high-grade dysplasia on the first specimen, abnormal blood liver tests.

IPMN: Intraductal papillary mucinous neoplasms; CA 19.9: cancer antigen 19.9; CECT: contrast-enhanced CT; WF: worrisome findings; HRS: high risk stigmata

**Table 2.** Complementary subset of radiomic features in the training cohort and the external validation cohort presenting statistically significant differences between benign and malignant groups. The adjusted p-value, AUC, optimal cut-off, accuracy, sensitivity, and specificity are provided for the training cohort.

| Features | Training Cohort | | | | | | External Validation Test Cohort | | |
|---|---|---|---|---|---|---|---|---|---|
| | p-value adj. | AUC | Cutt-off | Accuracy | Sensitivity | Specificity | Accuracy | Sensitivity | Specificity |
| firstorder_Energy | 0.00 | 0.75 | 19284480.50 | 0.69 | 0.65 | 0.74 | 0.58 | 0.48 | 0.70 |
| firstorder_TotalEnergy | 0.00 | 0.75 | 24105600.63 | 0.69 | 0.65 | 0.74 | 0.62 | 0.47 | 0.81 |
| gldm_SmallDependenceLowGrayLevelEmphasis | 0.00 | 0.75 | 0.00 | 0.71 | 0.75 | 0.66 | 0.54 | 0.41 | 0.70 |
| glszm_SmallAreaLowGrayLevelEmphasis | 0.00 | 0.74 | 0.00 | 0.71 | 0.79 | 0.62 | 0.54 | 0.44 | 0.67 |
| glrlm_ShortRunLowGrayLevelEmphasis | 0.00 | 0.74 | 0.00 | 0.70 | 0.73 | 0.66 | 0.50 | 0.36 | 0.68 |
| glrlm_LowGrayLevelRunEmphasis | 0.00 | 0.74 | 0.00 | 0.70 | 0.73 | 0.67 | 0.51 | 0.37 | 0.68 |
| glszm_LowGrayLevelZoneEmphasis | 0.00 | 0.74 | 0.00 | 0.71 | 0.80 | 0.60 | 0.53 | 0.44 | 0.65 |
| gldm_LowGrayLevelEmphasis | 0.00 | 0.74 | 0.00 | 0.70 | 0.73 | 0.67 | 0.51 | 0.37 | 0.68 |
| glrlm_LongRunLowGrayLevelEmphasis | 0.00 | 0.73 | 0.00 | 0.70 | 0.77 | 0.61 | 0.52 | 0.42 | 0.65 |
| glcm_Idmn | 0.00 | 0.73 | 0.97 | 0.69 | 0.70 | 0.69 | 0.50 | 0.56 | 0.42 |
| glcm_Idn | 0.00 | 0.73 | 0.89 | 0.69 | 0.67 | 0.71 | 0.50 | 0.55 | 0.44 |
| glcm_JointEntropy | 0.00 | 0.72 | 10.37 | 0.67 | 0.56 | 0.81 | 0.45 | 0.23 | 0.74 |
| gldm_DependenceEntropy | 0.00 | 0.72 | 6.41 | 0.65 | 0.51 | 0.81 | 0.42 | 0.15 | 0.75 |
| glcm_Correlation | 0.00 | 0.72 | 0.41 | 0.68 | 0.75 | 0.61 | 0.50 | 0.62 | 0.35 |
| shape_MeshVolume | 0.00 | 0.72 | 5112.34 | 0.69 | 0.82 | 0.52 | 0.62 | 0.70 | 0.53 |
| shape_VoxelVolume | 0.00 | 0.71 | 7002.50 | 0.68 | 0.72 | 0.62 | 0.64 | 0.68 | 0.58 |
| glrlm_RunLengthNonUniformity | 0.00 | 0.71 | 5162.89 | 0.68 | 0.72 | 0.63 | 0.62 | 0.64 | 0.58 |
| glszm_LargeAreaLowGrayLevelEmphasis | 0.00 | 0.71 | 0.00 | 0.68 | 0.61 | 0.75 | 0.45 | 0.22 | 0.75 |
| glszm_SizeZoneNonUniformity | 0.00 | 0.71 | 3785.52 | 0.68 | 0.72 | 0.63 | 0.61 | 0.64 | 0.56 |
| gldm_DependenceNonUniformity | 0.00 | 0.71 | 3508.83 | 0.68 | 0.72 | 0.63 | 0.61 | 0.64 | 0.56 |
| shape_SurfaceVolumeRatio | 0.00 | 0.70 | 0.61 | 0.68 | 0.77 | 0.57 | 0.61 | 0.67 | 0.53 |
| ngtdm_Coarseness | 0.00 | 0.70 | 0.00 | 0.67 | 0.82 | 0.50 | 0.63 | 0.73 | 0.51 |
| glrlm_GrayLevelNonUniformity | 0.00 | 0.70 | 138.88 | 0.68 | 0.73 | 0.61 | 0.64 | 0.71 | 0.54 |
| glszm_GrayLevelNonUniformity | 0.00 | 0.70 | 120.90 | 0.68 | 0.73 | 0.61 | 0.63 | 0.70 | 0.54 |
| glszm_ZoneEntropy | 0.00 | 0.70 | 6.01 | 0.67 | 0.73 | 0.58 | 0.45 | 0.38 | 0.54 |
| gldm_GrayLevelNonUniformity | 0.00 | 0.70 | 144.44 | 0.68 | 0.73 | 0.61 | 0.64 | 0.71 | 0.54 |
| shape_SurfaceArea | 0.00 | 0.70 | 3647.55 | 0.66 | 0.74 | 0.55 | 0.65 | 0.67 | 0.61 |
| glcm_MaximumProbability | 0.00 | 0.70 | 0.00 | 0.66 | 0.48 | 0.88 | 0.45 | 0.19 | 0.79 |
| glcm_JointEnergy | 0.00 | 0.69 | 0.00 | 0.66 | 0.54 | 0.80 | 0.46 | 0.21 | 0.79 |
| gldm_LargeDependenceLowGrayLevelEmphasis | 0.00 | 0.69 | 0.00 | 0.67 | 0.65 | 0.69 | 0.44 | 0.23 | 0.70 |
| shape_LeastAxisLength | 0.00 | 0.69 | 13.25 | 0.67 | 0.85 | 0.44 | 0.61 | 0.78 | 0.39 |
| shape_Maximum2DDiameterColumn | 0.00 | 0.68 | 48.62 | 0.65 | 0.63 | 0.67 | 0.62 | 0.55 | 0.70 |
| glcm_SumEntropy | 0.00 | 0.68 | 6.07 | 0.65 | 0.70 | 0.60 | 0.44 | 0.27 | 0.65 |
| gldm_LargeDependenceHighGrayLevelEmphasis | 0.00 | 0.67 | 2486.59 | 0.65 | 0.51 | 0.83 | 0.45 | 0.21 | 0.75 |

| | | | | | | | | | |
|---|---|---|---|---|---|---|---|---|---|
| **shape_MinorAxisLength** | 0.00 | 0.67 | 28.72 | 0.62 | 0.54 | 0.72 | 0.62 | 0.51 | 0.77 |
| **firstorder_Maximum** | 0.00 | 0.67 | 126.50 | 0.68 | 0.78 | 0.56 | 0.51 | 0.45 | 0.58 |
| **glszm_LargeAreaHighGrayLevelEmphasis** | 0.00 | 0.67 | 2059.42 | 0.65 | 0.49 | 0.83 | 0.44 | 0.18 | 0.77 |
| **ngtdm_Busyness** | 0.00 | 0.66 | 0.19 | 0.67 | 0.87 | 0.41 | 0.60 | 0.85 | 0.28 |
| **shape_Maximum2DDiameterSlice** | 0.00 | 0.66 | 41.32 | 0.64 | 0.66 | 0.61 | 0.57 | 0.55 | 0.60 |
| **glrlm_RunEntropy** | 0.00 | 0.66 | 5.60 | 0.65 | 0.70 | 0.57 | 0.43 | 0.27 | 0.63 |
| **firstorder_Range** | 0.00 | 0.65 | 192.50 | 0.63 | 0.66 | 0.60 | 0.48 | 0.29 | 0.72 |
| **glcm_Autocorrelation** | 0.00 | 0.65 | 1236.73 | 0.64 | 0.53 | 0.78 | 0.45 | 0.21 | 0.75 |
| **shape_Maximum3DDiameter** | 0.00 | 0.65 | 57.12 | 0.65 | 0.68 | 0.61 | 0.60 | 0.59 | 0.61 |
| **glcm_JointAverage** | 0.00 | 0.65 | 34.64 | 0.65 | 0.51 | 0.81 | 0.45 | 0.21 | 0.77 |
| **glcm_SumAverage** | 0.00 | 0.65 | 69.27 | 0.65 | 0.51 | 0.81 | 0.45 | 0.21 | 0.77 |
| **shape_Maximum2DDiameterRow** | 0.00 | 0.65 | 33.48 | 0.62 | 0.72 | 0.51 | 0.63 | 0.68 | 0.56 |
| **glcm_ClusterProminence** | 0.00 | 0.65 | 281367.25 | 0.66 | 0.72 | 0.57 | 0.43 | 0.29 | 0.61 |
| **ngtdm_Strength** | 0.00 | 0.65 | 2.22 | 0.65 | 0.76 | 0.51 | 0.62 | 0.75 | 0.44 |
| **glcm_ClusterTendency** | 0.00 | 0.65 | 303.10 | 0.65 | 0.71 | 0.57 | 0.43 | 0.29 | 0.61 |
| **glrlm_LongRunHighGrayLevelEmphasis** | 0.00 | 0.64 | 1422.50 | 0.64 | 0.56 | 0.73 | 0.45 | 0.23 | 0.74 |
| **firstorder_Skewness** | 0.00 | 0.64 | 0.23 | 0.63 | 0.63 | 0.63 | 0.60 | 0.58 | 0.63 |
| **glszm_HighGrayLevelZoneEmphasis** | 0.00 | 0.64 | 1398.57 | 0.63 | 0.50 | 0.79 | 0.43 | 0.15 | 0.79 |
| **glszm_SizeZoneNonUniformityNormalized** | 0.00 | 0.64 | 0.80 | 0.65 | 0.73 | 0.54 | 0.57 | 0.79 | 0.28 |
| **glrlm_HighGrayLevelRunEmphasis** | 0.01 | 0.64 | 1380.57 | 0.63 | 0.51 | 0.78 | 0.44 | 0.16 | 0.79 |
| **glszm_SmallAreaEmphasis** | 0.00 | 0.64 | 0.91 | 0.65 | 0.77 | 0.50 | 0.58 | 0.81 | 0.28 |
| **ngtdm_Complexity** | 0.01 | 0.64 | 9374.40 | 0.62 | 0.70 | 0.52 | 0.48 | 0.34 | 0.65 |
| **gldm_HighGrayLevelEmphasis** | 0.01 | 0.64 | 1374.76 | 0.63 | 0.51 | 0.78 | 0.45 | 0.18 | 0.79 |
| **gldm_SmallDependenceEmphasis** | 0.01 | 0.64 | 0.83 | 0.65 | 0.77 | 0.49 | 0.58 | 0.81 | 0.28 |
| **glrlm_ShortRunHighGrayLevelEmphasis** | 0.01 | 0.63 | 1343.93 | 0.62 | 0.51 | 0.77 | 0.44 | 0.16 | 0.79 |
| **glszm_ZonePercentage** | 0.01 | 0.63 | 0.88 | 0.64 | 0.72 | 0.54 | 0.55 | 0.77 | 0.28 |
| **gldm_DependenceNonUniformityNormalized** | 0.01 | 0.63 | 0.65 | 0.65 | 0.77 | 0.49 | 0.58 | 0.81 | 0.28 |
| **ngtdm_Contrast** | 0.01 | 0.63 | 0.32 | 0.59 | 0.42 | 0.78 | 0.59 | 0.67 | 0.49 |
| **glszm_LargeAreaEmphasis** | 0.01 | 0.63 | 1.45 | 0.64 | 0.79 | 0.47 | 0.58 | 0.82 | 0.28 |
| **glrlm_RunPercentage** | 0.01 | 0.63 | 0.97 | 0.64 | 0.79 | 0.47 | 0.58 | 0.82 | 0.28 |
| **glrlm_RunVariance** | 0.01 | 0.63 | 0.03 | 0.63 | 0.77 | 0.47 | 0.58 | 0.81 | 0.28 |
| **glszm_ZoneVariance** | 0.01 | 0.63 | 0.18 | 0.63 | 0.70 | 0.54 | 0.56 | 0.75 | 0.32 |
| **glrlm_LongRunEmphasis** | 0.01 | 0.63 | 1.10 | 0.64 | 0.78 | 0.47 | 0.58 | 0.81 | 0.28 |
| **glrlm_RunLengthNonUniformityNormalized** | 0.01 | 0.63 | 0.94 | 0.65 | 0.79 | 0.47 | 0.58 | 0.82 | 0.28 |
| **glrlm_ShortRunEmphasis** | 0.01 | 0.63 | 0.98 | 0.64 | 0.78 | 0.47 | 0.58 | 0.82 | 0.28 |
| **glszm_SmallAreaHighGrayLevelEmphasis** | 0.01 | 0.63 | 1304.59 | 0.62 | 0.48 | 0.79 | 0.44 | 0.15 | 0.81 |
| **gldm_LargeDependenceEmphasis** | 0.01 | 0.63 | 1.79 | 0.64 | 0.79 | 0.45 | 0.59 | 0.84 | 0.28 |
| **firstorder_Entropy** | 0.01 | 0.63 | 5.48 | 0.62 | 0.60 | 0.66 | 0.42 | 0.15 | 0.77 |
| **shape_MajorAxisLength** | 0.01 | 0.62 | 42.09 | 0.61 | 0.73 | 0.47 | 0.62 | 0.64 | 0.58 |
| **firstorder_90Percentile** | 0.01 | 0.62 | 82.80 | 0.62 | 0.60 | 0.64 | 0.46 | 0.29 | 0.68 |
| **glcm_SumSquares** | 0.01 | 0.62 | 118.42 | 0.62 | 0.58 | 0.66 | 0.43 | 0.16 | 0.77 |
| **gldm_DependenceVariance** | 0.02 | 0.62 | 0.24 | 0.63 | 0.84 | 0.39 | 0.59 | 0.86 | 0.25 |
| **gldm_SmallDependenceHighGrayLevelEmphasis** | 0.02 | 0.62 | 1190.71 | 0.61 | 0.47 | 0.78 | 0.44 | 0.15 | 0.81 |
| **glszm_GrayLevelNonUniformityNormalized** | 0.02 | 0.62 | 0.03 | 0.62 | 0.62 | 0.62 | 0.41 | 0.18 | 0.70 |
| **glrlm_GrayLevelNonUniformityNormalized** | 0.02 | 0.62 | 0.03 | 0.61 | 0.60 | 0.62 | 0.42 | 0.18 | 0.74 |
| **firstorder_Uniformity** | 0.02 | 0.62 | 0.03 | 0.61 | 0.58 | 0.64 | 0.44 | 0.19 | 0.75 |
| **firstorder_RootMeanSquared** | 0.03 | 0.61 | 49.96 | 0.61 | 0.60 | 0.62 | 0.48 | 0.36 | 0.65 |
| **firstorder_Variance** | 0.05 | 0.61 | 1018.4 | 0.62 | 0.70 | 0.52 | 0.39 | 0.22 | 0.61 |

| | | | | | | | | | |
|---|---|---|---|---|---|---|---|---|---|
| | | | 2 | | | | | | |
| **glrlm_GrayLevelVariance** | 0.05 | 0.61 | 114.52 | 0.62 | 0.70 | 0.52 | 0.39 | 0.22 | 0.61 |
| **gldm_GrayLevelVariance** | 0.05 | 0.61 | 113.23 | 0.62 | 0.70 | 0.52 | 0.39 | 0.22 | 0.61 |
| **glszm_GrayLevelVariance** | 0.05 | 0.61 | 119.15 | 0.62 | 0.70 | 0.53 | 0.39 | 0.22 | 0.61 |

**Table 3.** Cross-validation and external validation performance of models using only radiomic features and using radiomic features + clinical variables to differentiate between low-grade and high-grade pancreatic IPMN patients.

| | **Radiomics-only** | | **Radiomics + Surgical indication variables** | |
|---|---|---|---|---|
| | **CV mean [95% CI]** | **External Validation** | **CV mean [95% CI]** | **External Validation** |
| AUC | 0.80 [0.74 - 0.87] | 0.81 | 0.82 [0.76 - 0.88] | 0.77 |
| Acc | 0.75 [0.73 - 0.77] | 0.72 | 0.77 [0.74 - 0.79] | 0.67 |
| Se | 0.72 [0.68 - 0.75] | 0.68 | 0.74 [0.69 - 0.78] | 0.62 |
| Spe | 0.78 [0.75 - 0.81] | 0.76 | 0.79 [0.76 - 0.81] | 0.70 |
| PPV | 0.70 [0.68 - 0.73] | 0.68 | 0.71 [0.68 - 0.74] | 0.61 |
| NPV | 0.81 [0.79 - 0.83] | 0.76 | 0.83 [0.80 - 0.86] | 0.72 |
| MCC | 0.50 [0.46 - 0.54] | 0.43 | 0.53 [0.48 - 0.58] | 0.33 |

(CV) : cross-validation ; (AUC) : Area Under the receiver operating characteristic Curve ; (Acc) : accuracy ; (Se) : sensitivity ; (Spe) : specificity ; (PPV) : positive predictive values ; (NPV) : negative predictive values; (MCC) : Matthews Correlation Coefficient

**Table 4.** Cross-validation and external validation performance of models using only radiomic features and using radiomic features + clinical variables for the differentiation between patients with low-grade, high-grade, and invasive pancreatic IPMNs.

| | **Radiomics-only** | | | | **Radiomics + Surgical indication variables** | | | |
|---|---|---|---|---|---|---|---|---|
| | **CV mean [95% CI]** | **External Validation** | | | **CV mean [95% CI]** | **External Validation** | | |
| AUC | 0.81 [na] | 0.73 | | | 0.81 [na] | 0.73 | | |
| Acc | 0.72 [0.70 - 0.75] | 0.62 | | | 0.75 [0.73 - 0.76] | 0.63 | | |
| | | Low-Grade | High-Grade | Invasive | | Low-Grade | High-Grade | Invasive |
| Se | 0.72 [0.69 - 0.75] | 0.67 | 0.63 | 0.53 | 0.74 [0.73 - 0.76] | 0.63 | 0.65 | 0.59 |
| Spe | 0.86 [0.84 - 0.87] | 0.67 | 0.86 | 0.87 | 0.87 [0.86 - 0.87] | 0.71 | 0.83 | 0.88 |
| PPV | 0.75 [0.72 - 0.77] | 0.60 | 0.68 | 0.59 | 0.77 [0.75 - 0.78] | 0.62 | 0.63 | 0.63 |
| NPV | 0.86 [0.85 - 0.87] | 0.73 | 0.83 | 0.85 | 0.87 [0.86 - 0.88] | 0.72 | 0.84 | 0.8 |
| MCC | 0.58 [0.54 - 0.62] | 0.41 | | | 0.61 [0.59 - 0.63] | 0.43 | | |

CV : cross-validation ; AUC : Area Under the receiver operating characteristic Curve ; Acc : accuracy ; Se ; sensitivity ; PPV : positive predictive values ; NPV : negative predictive values; MCC : Matthews Correlation Coefficient

**Table 5.** Cross-validation and external validation performance of models using only radiomic features and using radiomic features + clinical variables to differentiate between patients with

high-grade and invasive pancreatic IPMNs.

| | Radiomics-only | | Radiomics + Surgical indication variables | |
|---|---|---|---|---|
| | **CV mean [95% CI]** | **External Validation** | **CV mean [95% CI]** | **External Validation** |
| AUC | 0.92 [0.88 - 0.96] | 0.91 | 0.92 [0.87 - 0.96] | 0.92 |
| Acc | 0.85 [0.83 - 0.88] | 0.83 | 0.87 [0.84 - 0.89] | 0.83 |
| Se | 0.81 [0.76 - 0.85] | 0.69 | 0.82 [0.78 - 0.86] | 0.69 |
| Spe | 0.89 [0.86 - 0.91] | 0.95 | 0.90 [0.88 - 0.92] | 0.95 |
| PPV | 0.85 [0.82 - 0.88] | 0.92 | 0.86 [0.84 - 0.89] | 0.92 |
| NPV | 0.87 [0.84 - 0.89] | 0.79 | 0.88 [0.85 - 0.90] | 0.79 |
| MCC | 0.71 [0.66 - 0.75] | 0.67 | 0.73 [0.68 - 0.78] | 0.67 |

CV : cross-validation ; AUC : Area Under the receiver operating characteristic Curve ; Acc : accuracy ; Se sensitivity ; Spe : Specificity ; PPV : positive predictive values ; NPV : negative predictive values; MCC : Matthews Correlation Coefficient

**Table 6.** Cross-validation and external validation performance of models using only radiomic features and using radiomic features + clinical variables to differentiate between low-grade and high-grade pancreatic BD-IPMN patients.

.

| | Radiomics-only | | Radiomics + Surgical indication variables | |
|---|---|---|---|---|
| | **CV mean [95% CI]** | **External Validation** | **CV mean [95% CI]** | **External Validation** |
| AUC | 0.68 [0.55 - 0.81] | 0.58 | 0.68 [0.55 - 0.81] | 0.61 |
| Acc | 0.68 [0.63 - 0.72] | 0.70 | 0.50 [0.44 - 0.56] | 0.45 |
| Se | 0.58 [0.46 - 0.70] | 0.50 | 0.82 [0.73 - 0.90] | 0.75 |
| Spe | 0.71 [0.63 - 0.79] | 0.76 | 0.40 [0.34 - 0.47] | 0.36 |
| PPV | 0.47 [0.36 - 0.59] | 0.40 | 0.31 [0.27 - 0.35] | 0.27 |
| NPV | 0.86 [0.82 - 0.90] | 0.83 | 0.87 [0.81 - 0.94] | 0.82 |
| MCC | 0.31 [0.20 - 0.41] | 0.24 | 0.20 [0.09 - 0.30] | 0.10 |

CV : cross-validation ; AUC : Area Under the receiver operating characteristic Curve ; Acc : accuracy ; Se : sensitivity ; Spe : specificity ; PPV : positive predictive values ; NPV : negative predictive values; MCC : Matthews Correlation Coefficient

**Table 7.** Cross-validation and external validation performance of models using only radiomic features and using radiomic features + clinical variables to differentiate between patients with low-grade, high-grade, and invasive pancreatic IPMNs.

| | Radiomics-only | | | | Radiomics + Surgical indication variables | | | |
|---|---|---|---|---|---|---|---|---|
| | **CV mean [95% CI]** | **External Validation** | | | **CV mean [95% CI]** | **External Validation** | | |
| AUC | 0.63 [0.05 - 0.05] | 0.7686 | | | 0.75 [0.06 - 0.06] | 0.70 | | |
| Acc | 0.51 [0.46 - 0.56] | 0.4615 | | | 0.51 [0.45 - 0.56] | 0.41 | | |
| Se | 0.36 [0.30 - 0.41] | Low-Grade | High-Grade | Invasive | 0.46 [0.38 - 0.54] | Low-Grade | High-Grade | Invasive |

| | | | | | | | | |
|---|---|---|---|---|---|---|---|---|
| | | 0.52 | 0.50 | 0.17 | | 0.36 | 0.75 | 0.17 |
| Spe | 0.74 [0.71 - 0.78] | 0.36 | 0.74 | 0.88 | 0.76 [0.72 - 0.80] | 0.71 | 0.42 | 0.97 |
| PPV | 0.33 [0.30 - 0.36] | 0.59 | 0.33 | 0.20 | 0.48 [0.37 - 0.58] | 0.6 | 0.25 | 0.50 |
| NPV | 0.70 [0.67 - 0.72] | 0.29 | 0.85 | 0.85 | 0.75 [0.71 - 0.79] | 0.38 | 0.87 | 0.86 |
| MCC | 0.17 [0.08 - 0.26] | 0.03 | | | 0.61 [0.59 - 0.63] | 0.43 | | |

CV : cross-validation ; AUC : Area Under the receiver operating characteristic Curve ; Acc : accuracy ; Se : sensitivity ; Spe : specificity ; PPV : positive predictive values ; NPV : negative predictive values; MCC : Matthews Correlation Coefficient

**Table 8.** Cross-validation and external validation performance of models using only radiomic features and using radiomic features + clinical variables to differentiate between patients with high-grade and invasive pancreatic BD-IPMNs.

| | **Radiomics-only** | | **Radiomics + Surgical indication variables** | |
|---|---|---|---|---|
| | **CV mean [95% CI]** | **External Validation** | **CV mean [95% CI]** | **External Validation** |
| AUC | 0.66 [0.41 - 0.91] | 0.85 | 0.73 [0.50 - 0.95] | 0.88 |
| Acc | 0.73 [0.56 - 0.90] | 0.71 | 0.80 [0.69 - 0.91] | 0.64 |
| Se | 0.29 [0.00 - 0.60] | 0.33 | 0.29 [0.00 - 0.60] | 0.17 |
| Spe | 0.87 [0.73 - 1.00] | 1.00 | 0.97 [0.90 - 1.00] | 1.00 |
| PPV | 0.50 [0.13 - 0.87] | 1.00 | 1.00 [1.00 - 1.00] | 1.00 |
| NPV | 0.71 [0.58 - 0.85] | 0.67 | 0.76 [0.66 - 0.87] | 0.62 |
| MCC | 0.10 [0.00 - 0.43] | 0.47 | 0.20 [0.00 - 0.47] | 0.32 |

CV : cross-validation ; AUC : Area Under the receiver operating characteristic Curve ; Acc : accuracy ; Se : sensitivity ; Spe : specificity ; PPV : positive predictive values ; NPV : negative predictive values; MCC : Matthews Correlation Coefficient

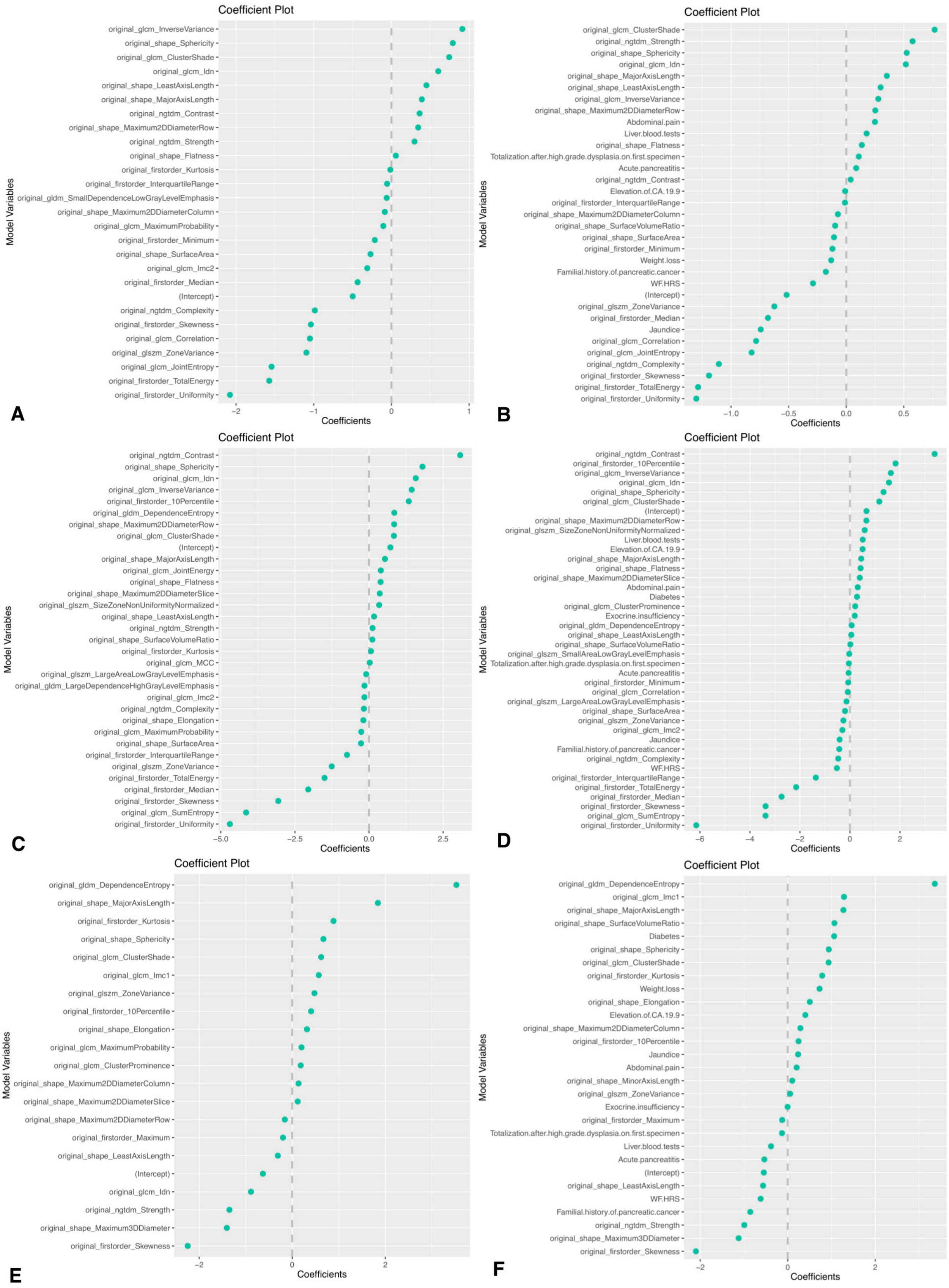


**Figure 1.** Coefficients of selected variables for (A) radiomics and (B) radiomics + surgical indications models for the differentiation between benign and malignant IPMNs. (C) and (D) provide the coefficients of selected variables for radiomics and radiomics + surgical indications models for the differentiation of low-grade and high-grade dysplasia IPMNs, while (E) and (F) show the

coefficients of radiomics and radiomics + surgical indications models for differentiation between high-grade and invasive IPMNs.

© 2020 by the authors. Licensee MDPI, Basel, Switzerland. This article is an open access article distributed under the terms and conditions of the Creative Commons Attribution (CC BY) license (http://creativecommons.org/licenses/by/4.0/).
